# Vacuum Ultraviolet (VUV) Light Photofunctionalization to Induce Human Oral Fibroblast Transmigration on Zirconia

**DOI:** 10.3390/cells12212542

**Published:** 2023-10-29

**Authors:** Toshikatsu Suzumura, Takanori Matsuura, Keiji Komatsu, Yoshihiko Sugita, Hatsuhiko Maeda, Takahiro Ogawa

**Affiliations:** 1 Weintraub Center for Reconstructive Biotechnology, Division of Regenerative and Reconstructive Sciences, UCLA School of Dentistry, Los Angeles, CA 90095-1668, USA; 2 Department of Oral Pathology/Forensic Odontology, School of Dentistry, Aichi Gakuin University, Nagoya 464-8650, Japan

**Keywords:** dental and orthopedic implants, titanium, hydrocarbon, implant pellicle, hydrophilic

## Abstract

Soft tissue adhesion and sealing around dental and maxillofacial implants, related prosthetic components, and crowns are a clinical imperative to prevent adverse outcomes of periodontitis and periimplantitis. Zirconia is often used to fabricate implant components and crowns. Here, we hypothesized that UV treatment of zirconia would induce unique behaviors in fibroblasts that favor the establishment of a soft tissue seal. Human oral fibroblasts were cultured on zirconia specimens to confluency before placing a second zirconia specimen (either untreated or treated with one minute of 172 nm vacuum UV (VUV) light) next to the first specimen separated by a gap of 150 µm. After seven days of culture, fibroblasts only transmigrated onto VUV-treated zirconia, forming a 2.36 mm volume zone and 5.30 mm leading edge. Cells migrating on VUV-treated zirconia were enlarged, with robust formation of multidirectional cytoplastic projections, even on day seven. Fibroblasts were also cultured on horizontally placed and 45° and 60° tilted zirconia specimens, with the latter configurations compromising initial attachment and proliferation. However, VUV treatment of zirconia mitigated the negative impact of tilting, with higher tilt angles increasing the difference in cellular behavior between control and VUV-treated specimens. Fibroblast size, perimeter, and diameter on day seven were greater than on day one exclusively on VUV-treated zirconia. VUV treatment reduced surface elemental carbon and induced superhydrophilicity, confirming the removal of the hydrocarbon pellicle. Similar effects of VUV treatment were observed on glazed zirconia specimens with silica surfaces. One-minute VUV photofunctionalization of zirconia and silica therefore promotes human oral fibroblast attachment and proliferation, especially under challenging culture conditions, and induces specimen-to-specimen transmigration and sustainable photofunctionalization for at least seven days.

## 1. Introduction

Zirconia is widely used in modern dental and maxillofacial prosthetics, including but not limited to dental implants, crowns, inlays, tissue bars, and implant abutments (connecting an implant and crown) [1,2,3,4,5,6,7,8,9,10,11,12,13,14,15,16,17,18,19,20,21,22,23,24,25]. All are placed transmucosally or mucosally with the expectation that gingival soft tissue will adhere, grow, and cover them to seal the zirconia interface and prevent bacterial invasion from the oral cavity. Nevertheless, periodontal disease and periimplantitis caused by bacterial infection are primary and increasing causes of tooth and implant failure [26,27,28,29,30,31,32,33,34,35,36,37,38,39]. Indeed, gingival tissue rarely fully adheres to zirconia or titanium surfaces in clinical settings.

The ultraviolent (UV)-light-mediated photofunctionalization of titanium has revolutionized our understanding of bone–titanium integration, or osseointegration [40,41,42,43,44,45,46,47,48,49,50]. UV photofunctionalization describes the treatment of titanium surfaces with UV light immediately before use to decarbonize the surface [40,42,44,51,52,53,54,55,56,57,58,59,60,61,62,63,64,65,66]. Specifically, UV treatment removes a pellicle of hydrocarbons from titanium surfaces that accumulates over time [41,48,67,68,69,70,71,72,73,74,75,76,77]. As a result, hydrophobic, hydrocarbon-contaminated titanium surfaces become superhydrophilic, and decarbonized, pellicle-free titanium surfaces recruit more osteogenic cells [67], promote and strengthen cellular attachment [78,79], and the density/proliferation of cells [80] and other cellular phenotypes is increased [50]. Blood and protein recruitment is also enhanced around pellicle-free, superhydrophilic implants in in silico simulation. In vivo benefits include two-time increase in bone volume [65] and a near 100% bone–implant contact (cf. 53% in controls) [67], and thereby faster and stronger osseointegration [54,56,60,63,67,81,82]. These biological effects have the measurable clinical benefit of accelerating the healing time before establishing the implant anchor [56,59], mitigating risk factors that might complicate implant therapy [83,84] and expanding the therapeutic indications. However, the effect of UV treatment on fibroblast function remains incompletely understood [85,86,87], and whether photofunctionalization occurs on zirconia has rarely been explored.

Next-generation UV photofunctionalization, or vacuum UV (VUV) photofunctionalization [88,89], may further enhance material bioactivity. A limitation of classical UV photofunctionalization protocols was the UV exposure time—12 or 20 min, or longer up to 48 h—needed to remove the hydrocarbon pellicle [46,66,67,83,90,91,92]. Since implants must be treated with UV immediately prior to use to ensure efficacy, these long treatment times were a significant barrier to using the technique during surgery. Recently, high-energy UV light (wavelength < 200 nm), or VUV, was introduced for photofunctionalization [88,89]. VUV can maximally decompose and remove hydrocarbons in the liquid and on titanium surfaces in one minute, drastically improving the exposure time. Further clarifying the potential of VUV as an energy source for photofunctionalization is urgently required.

The objective of this study was to examine the effect of VUV light treatment of zirconia on the behavior and function of human oral fibroblasts. To create a biological seal, oral/gingival fibroblasts must interact with the three-dimensional contours of zirconia surfaces and, if possible, also extend and migrate over the zirconia surfaces. Therefore, here, we examined the potential of VUV-treated zirconia to induce distinct fibroblast behaviors in vitro, especially under challenging culture conditions, mimicking the complexity of the oral environment. For clinical correlation, we also examined glazed zirconia surfaces, a technique widely used for zirconia crowns and other dental restorations, in addition to regular milled zirconia surfaces.

## 2. Materials and Methods

### 2.1. Zirconia Specimen Preparation and Characterization

Zirconia specimens in rectangular plate form (14 × 6 mm, 2 mm thick) were milled from a zirconia disk (Aurident, Fullerton, CA, USA) and sintered at 1530 °C for 120 min. Glazed specimens were prepared by coating zirconia specimens with silica glass paste (InSync Glase Paste Fluor, Jensen Industries Inc., North Haven, CT, USA) followed by heating at 830 °C. All specimens were prepared by World Lab USA (Irvine, CA, USA).

UV treatment was performed at room temperature using a vacuum UV (VUV) light (172 nm VUV, 60 mW/cm^2^; DIO Implant, Busan, Korea) for 1 min. Surface morphology was examined via scanning electron microscopy (SEM; Nova 230 Nano SEM, FEI, Hillsboro, OR, USA). In addition, the following roughness parameters were quantified via atomic force microscopy (AFM; VN-8010, Keyence, Osaka, Japan): average roughness (Ra), peak-to-valley roughness (Rz), skewness (Rsk), kutosis (Rku), and mean width of profile elements (Rsm). The hydrophilicity/hydrophobicity of zirconia surfaces with and without VUV treatment was evaluated by measuring the contact angle of 3 µL ddH_2_O, as previously optimized [93].

### 2.2. Cell Culture

Primary human oral fibroblasts (ScienCell Research Laboratories, Carlsbad, CA, USA) were cultured, as reported elsewhere [94,95,96], in a company-recommended medium supplemented with 5% fetal bovine serum (FBS), 1% fibroblast growth supplement-2 (ScienCell Research Laboratories), and 1% penicillin/streptomycin solution. At 80% confluence, the cells were detached using 0.05% trypsin-EDTA solution and seeded onto each test zirconia specimen placed in a well (20 mm diameter) of 12-well culture plates at a density of 4 × 10^4^ cells/well. The culture medium was renewed every three days. Zirconia specimens were placed in the wells either horizontally or tilted to 45° or 60°, with a piece of titanium plate used as a mounting substrate. To determine the ability of fibroblasts to migrate from specimen to specimen, a transmigration assay was developed by culturing fibroblasts to confluency on a zirconia specimen and then placing a second new specimen next to the first with a physical intimate contact. The culture continued to day 7.

### 2.3. Quantification of Attached and Proliferated Cells

The number of fibroblasts attaching to zirconia specimens during the first day of culture was determined by counting the cells in fluorescence microscopy images. The cells were fixed in 10% formalin, permeabilized with 0.5% TritonX-100, blocked with 1% BSA, and then stained with rhodamine–phalloidin (actin filaments, red; Molecular Probes, Eugene, OR, USA) for actin filaments. Cell proliferation was evaluated by measuring cell density on days 2 and 4 using the water-soluble tetrazolium salt (WST-1)-based colorimetric assay, as reported elsewhere [78,79,97]. In addition, BrdU incorporation into DNA (Roche Applied Science, Mannheim, Germany) was measured to confirm cell proliferation on day 4, as reported elsewhere [98].

### 2.4. Cell Morphology, Morphometry, and Transmigration

The spreading behavior of fibroblasts seeded onto zirconia specimens was examined via fluorescence microscopy. On day 1, the cells were fixed and stained using the method above and evaluated for cell area, perimeter, and Feret’s diameter using ImageJ (NIH, Bethesda, MD, USA).

To assess the transmigration, stained cells on the second specimen were evaluated via fluorescence microscopy on day 7. To capture the largest area, the lowest magnification images (×40) were taken and compiled to cover the entire depth of a specimen (6 mm). The images were obtained along the center line of the rectangular specimen, beginning at the middle of the 14 mm long side of the rectangle. Fibroblast migration was measured in two different ways: volume zone (the extension of confluent area formed by migrated cells) and leading edge (a cluster of cells at the leading edge of migration).

### 2.5. Collagen Production

Fibroblast collagen production was measured on day 4 via picrosirius red staining (Polysciences Inc., Warrington, PA, USA), as reported elsewhere [95,96]. Prior to staining, the cells were washed with PBS and fixed in 10% formaldehyde for 5 min. Subsequently, the cells were stained with 0.1% picrosirius red solution for 60 min at room temperature, after which 0.1 N sodium hydroxide was added for 60 min to elute the bound dye. The supernatant was measured at an absorbance of 550 nm using a microplate reader.

### 2.6. Gene Expression

On day 4 of culture, the specimens were collected. Total RNA was extracted using TRIzol reagent (Life Technologies, Carlsbad, CA, USA), and the RNA was purified using the Direct-zol RNA MiniPrep kit (Zymo Research, Irvine, CA, USA) according to the manufacturers’ protocols and established methods [99,100,101]. The RNA was quantified with a NanoDrop One instrument (Thermo Fisher Scientific, Waltham, MA, USA). The RNA was reverse transcribed with a SuperScript VILO cDNA Synthesis kit (Invitrogen, Carlsbad, CA, USA). Real-time quantitative polymerase chain reaction (PCR) was performed with the QuantStudioTM 3 Real-Time PCR System (Applied Biosystems, Waltham, MA, USA) to quantify mRNA expression of collagen type I, fibronectin, and integrin alpha 3. Gene expression levels were normalized to glyceraldehyde-3-phosphate dehydrogenase (Gapdh) expression.

### 2.7. Statistical Analysis

All cell culture assays were performed in triplicate (n = 3), except for cytomorphometry, which was conducted on twenty cells (n = 20). Quantitative surface analysis was conducted on six random areas of zirconia specimens (n = 6). Surface chemical assessment via XPS and contact angle measurements were undertaken on three different specimens (n = 3). Differences between the control and VUV-treated groups were assessed with *t*-tests; *p*-values < 0.05 were considered statistically significant.

## 3. Results

### 3.1. Surface Characteristics of Zirconia Specimens with or without VUV Treatment

**Figure 1:**
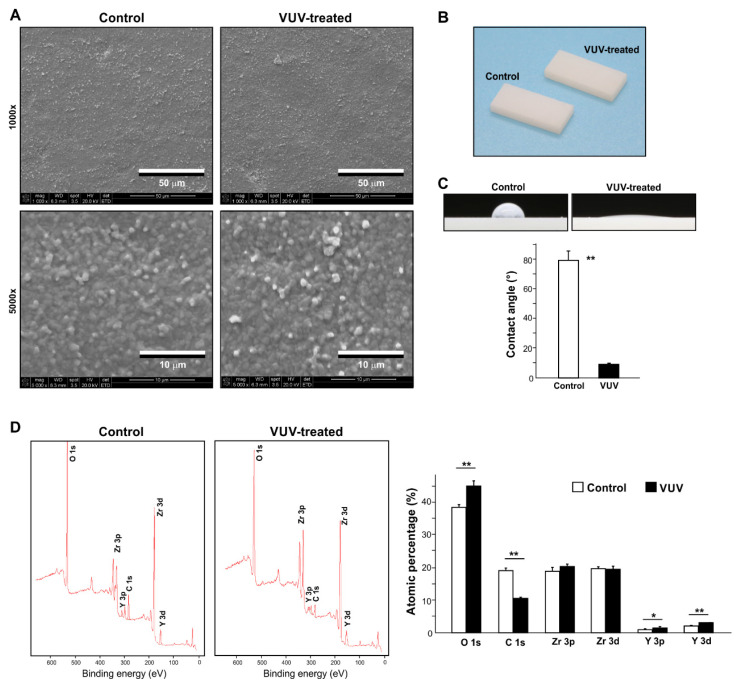
Surface characterization of milled zirconia surfaces. (**A**) SEM images of the milled zirconia specimens used in this study. Images before and after one-minute vacuum UV (VUV) light treatment are shown. (**B**) Photographs of zirconia specimens with and without VUV treatment. (**C**) Side-view images of 3 mL H_2_O placed on zirconia specimens with or without VUV treatment and a histogram showing the calculated contact angles. (**D**) Chemical element assessment of zirconia specimens. XPS spectrum for control and VUV-treated surfaces together with the quantified elemental percentages are shown. * *p* < 0.05, ** *p* < 0.01, statistically different between the control and VUV-treated groups.

The SEM images revealed rough submicron nodules all over the surface of the milled zirconia specimens (Figure 1A). There was no difference between the control and VUV-treated specimens. Photographs of the specimens did not show any change in color after VUV treatment (Figure 1B). Hydrophobic zirconia specimens became superhydrophilic (θ < 10°) after VUV treatment (Figure 1C).

X-ray photoelectron spectroscopy (XPS) was next used to detect the elements present on zirconia specimens, i.e., oxygen, carbon, zirconium, and yttrium (Figure 1D). There was a significant reduction in the atomic percentage of carbon from 18.9% to 10.7% after VUV treatment. Accordingly, oxygen and yttrium increased.

**Figure 2:**
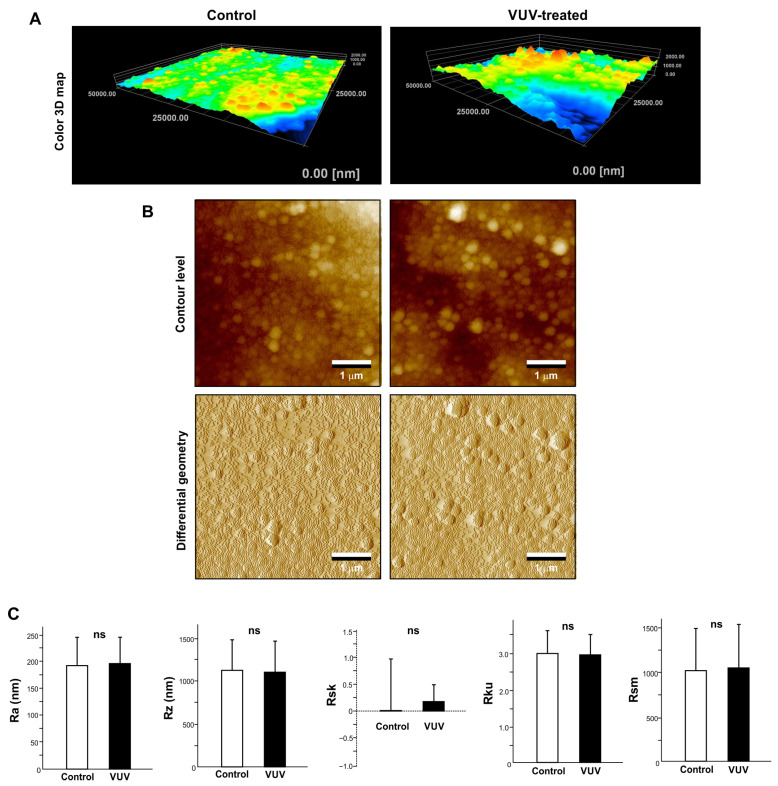
Surface topography of milled zirconia. Three-dimensional color maps (**A**), contour level and differential geometry two-dimensional (**B**) images of milled zirconia specimens via AFM. (**C**) Histograms of surface roughness parameters calculated via AFM. ns: not significant difference between the control and VUV-treated groups.

To further examine any VUV treatment-induced changes in surface topography, three-dimensional imaging and quantitative roughness analysis were performed via AFM. Three-dimensional color maps confirmed that the control and VUV-treated zirconia specimens had similar surface topographies (Figure 2A). Contour-level and differential geometry two-dimensional images were also similar regardless of VUV exposure (Figure 2B). There were no significant differences in roughness parameters between the control and VUV-treated specimens (Figure 2C).

### 3.2. Behavior and Function of Human Oral Fibroblasts on Zirconia with or without VUV Treatment

**Figure 3:**
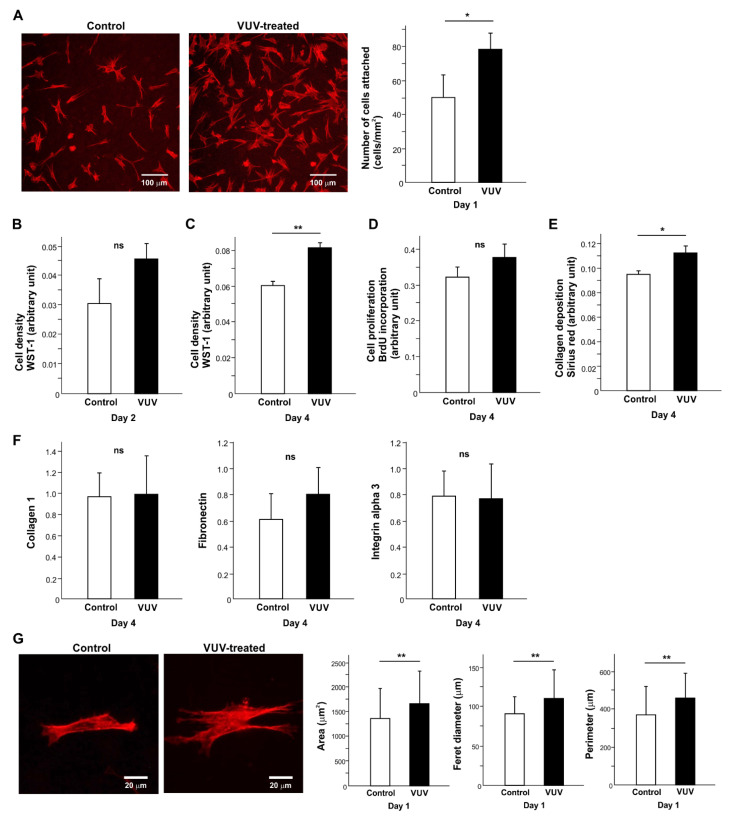
Fibroblast behavior and function on milled zirconia with or without VUV treatment. (**A**) The number of fibroblasts attaching to zirconia during the first day of culture. Representative fluorescence microscopy images and the number of attached cells counted on the images are shown. Cell density evaluated via WST-1 assay on days 2 (**B**) and 4 (**C**). (**D**) Cell proliferation evaluated via BrdU incorporation into DNA on day 4. (**E**) Collagen deposition evaluated on day 4. (**F**) The level of gene expression evaluated via real-time PCR. (**G**) Cytomorphometry. High magnification representative microscopic images of fibroblasts and measured metrics. * *p* < 0.05, ** *p* < 0.01, statistically different between the control and VUV-treated groups. ns: not significant.

We next examined how human oral fibroblasts react to milled zirconia specimens with or without VUV treatment. The cells were seeded on zirconia specimens placed horizontally in 12-well cell culture dish wells. Significantly more (~40%) cells attached to VUV-treated zirconia during the first day of culture than to untreated zirconia (Figure 3A).

To evaluate the rate of cell proliferation, cell density was measured on days 2 and 4 of culture (Figure 3B,C). There were more cells on VUV-treated zirconia than on control on day 4. While BrdU incorporation into DNA was not significantly different between the two groups (Figure 3D), collagen production was significantly higher in cells grown for 4 days on VUV-treated zirconia than on controls (Figure 3E). The gene expression of collagen 1, fibronectin, and integrin alpha 3 was not significantly modulated by VUV treatment (Figure 3F).

The spreading behavior was different on treated and untreated surfaces, with cells spreading on larger VUV-treated surfaces with robust cytoplasmic projections, as shown via fluorescence microscopy and cytomorphometric analysis of cell area, Ferret diameter, and perimeter (Figure 3G).

### 3.3. Fibroblast Attachment and Growth on VUV-Treated Zirconia under Challenging Conditions and Transmigration across Specimens

**Figure 4:**
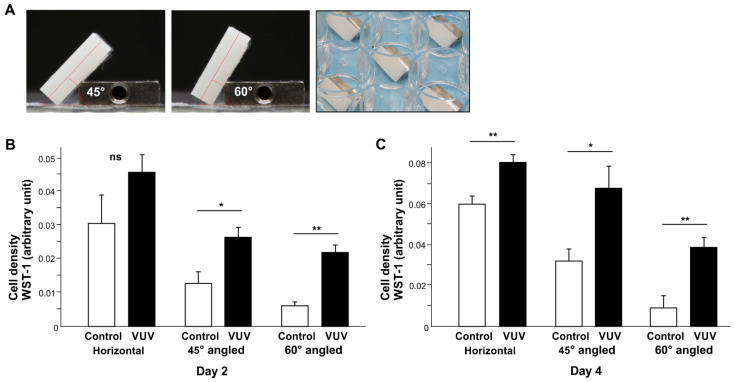
Fibroblast attachment and growth on zirconia specimens under challenging conditions. (**A**) Photographs of zirconia specimens placed in a well of a polystyrene culture dish at 45° and 60°. Cell density on zirconia specimens evaluated via the WST-1 assay on days 2 (**B**) and 4 (**C**). * *p*  <  0.05, ** *p*  <  0.01, statistically different between the control and VUV-treated groups. ns: not significant.

We next created challenging conditions for cell growth to mimic the clinical environment. Zirconia specimens were placed obliquely in the wells of culture dishes at 45° and 60° (Figure 4A) and cell density was evaluated on days 2 and 4 (Figure 4B,C). Specimen tilting negatively affected the cell density in an angle-dependent manner, with 60° producing the lowest cell density. On control specimens, there was an 80% reduction on 60° angled specimens compared with horizontal specimens both on days 2 and 4. VUV treatment increased the cell density on angled specimens more than on horizontal specimens, with an even greater difference on 60° specimens than on 45° specimens. Of note, the cell density on 45° VUV-treated zirconia was equivalent to that on horizontal control zirconia. Even on 60° specimens, the negative effect of specimen tilting was significantly mitigated by VUV treatment.

**Figure 5:**
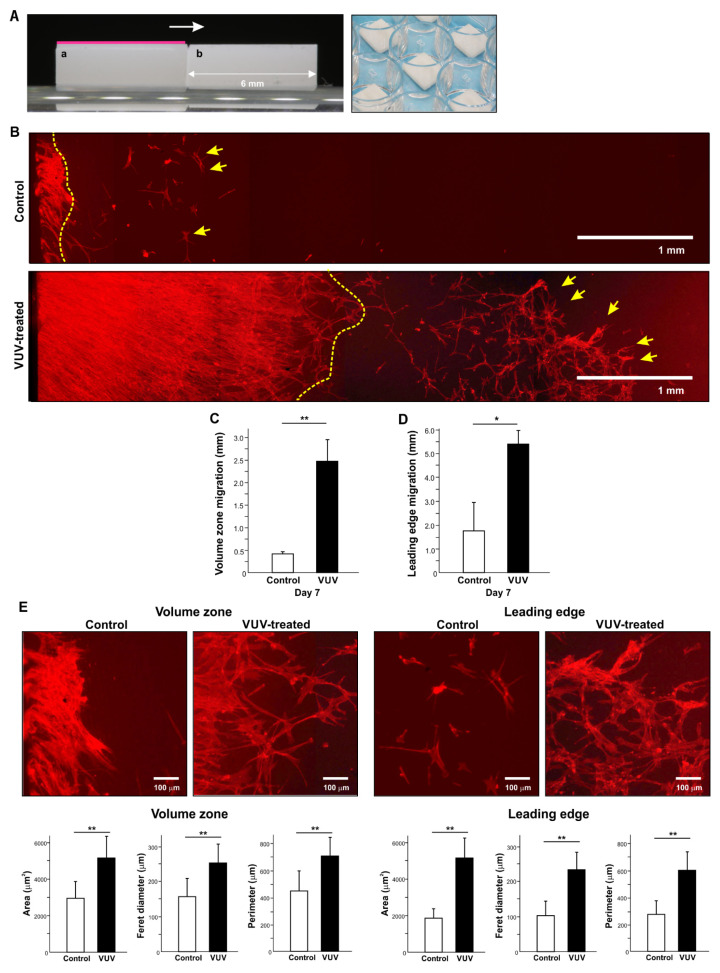
Transmigration of fibroblasts across specimens. (**A**) Culture setting with two zirconia specimens placed side-by-side. Fibroblasts were grown to confluency in advance on the first specimen (red line on specimen a), and then a new specimen with or without VUV treatment (specimen b) was placed next to the first specimen to allow for transmigration from specimen a to b. The photograph shows the two juxtaposed specimens (right panel). Note the 150 µm gap between specimens. (**B**) Representative fluorescent microscopy images of milled zirconia specimens with or without VUV treatment showing significant differences in the extent of cellular migration across specimens. Migration was initiated from the left side of the images. The yellow dotted line denotes the volume zone of fibroblast migration. Yellow arrows denote the cluster or colony of fibroblasts forming the leading edge of migration. (**C**) Maximum migration was defined as the extent of migration by fibroblasts at the leading edge. (**D**) Volume-zone migration was defined as the extent of migration formed by confluent fibroblasts. (**E**) Magnified microscopic images from the volume zone and leading edge of fibroblast migration on control and VUV-treated zirconia. Cytomorphometry results are also shown. * *p*  <  0.05, ** *p*  <  0.01, statistically different between the control and VUV-treated groups.

We next determined the potential of fibroblasts to migrate across zirconia specimens. First, we cultured fibroblasts to confluency on milled zirconia. Then, the specimen with attached cells was transferred to a new culture well with a second, brand-new specimen placed alongside (Figure 5A), expecting the cells to migrate from the original specimen to the adjacent new specimen (in the direction indicated by an arrow in Figure 5A). Due to the bevel on the edges of specimens made during milling, there was a 150 µm gap between the two specimens. We compared the new zirconia specimens with or without VUV treatment.

A cluster of fibroblasts migrated to the control zirconia surface, with the reach of migration being limited to the contact area of the new specimen (yellow dotted line in the top image, Figure 5B), with a few cells appearing at remote areas (arrows in the top image, Figure 5B). By contrast, there was more extensive and denser fibroblast migration on VUV-treated surfaces (yellow dotted line in the bottom image, Figure 5B). In addition, a significant number of cells migrated and colonized distant areas of VUV-treated surfaces (arrows in the bottom image, Figure 5B). The average migration volume zone of confluent cells was 0.41 mm and 2.26 mm for control and VUV-treated surfaces, respectively, representing a difference of six times (Figure 5C). The maximum migration, defined as the leading-edge migration, was 1.83 mm and 5.30 mm for control and VUV-treated surfaces, respectively, a three-time difference (Figure 5D). Cytomorphology and cytomorphometry of each migration zone and leading edge showed that cells were significantly larger with a more advanced development of cytoplasmic protrusions on VUV-treated zirconia (Figure 5E). In addition, these cytomorphometric parameters on day 7 were greater than those on day 1 only on VUV-treated zirconia but not on control zirconia.

### 3.4. Effects of VUV Treatment on Glazed Zirconia

We next attempted to generalize the observed biological effects by testing zirconia with a different surface finish. We chose glazed zirconia because porcelain glazing is commonly used to finish zirconia crowns and other dental restorations.

**Figure 6:**
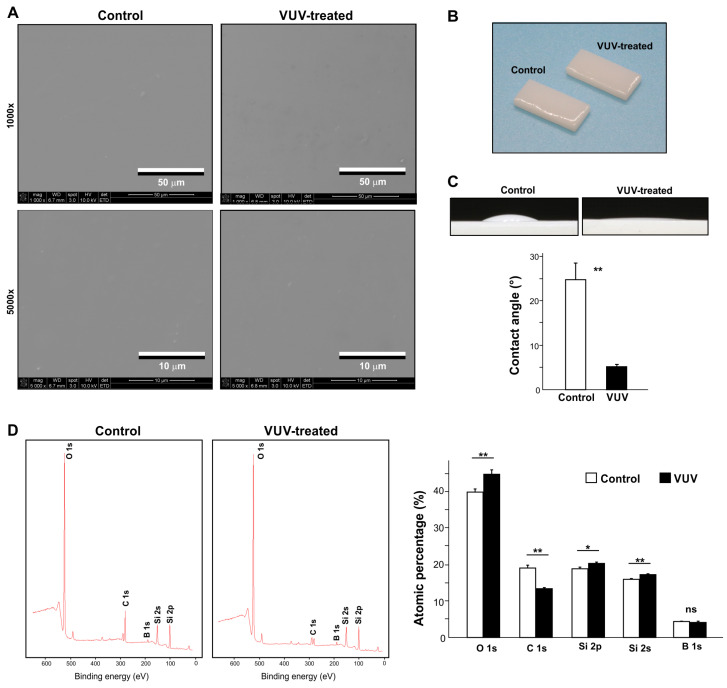
Surface characterization of glazed zirconia specimens. (**A**) SEM images of glazed zirconia specimens used in this study. Images before and after one-minute VUV light treatment are presented. (**B**) Photographs of the specimens with and without VUV treatment. (**C**) Side-view images of 3 mL H_2_O placed on the specimens with or without VUV treatment and a histogram showing the calculated contact angles. (**D**) Chemical element assessment of the glazed specimens. XPS spectrum for control and VUV-treated surfaces, as well as the quantified atomic percentages, are shown. * *p*  <  0.05, ** *p*  <  0.01, statistically different between the control and VUV-treated groups. ns: not significant.

SEM observation showed that glazed zirconia specimens were smoother than milled zirconia, with no detectable topography nor roughness at the micron scale (Figure 6A) and no morphological change after VUV treatment. Photographs again showed no surface differences or color alterations after VUV treatment (Figure 6B). VUV treatment induced the expected physicochemical changes on glazed zirconia, with the contact angle significantly decreasing to <10° (Figure 6C). XPS analysis revealed that the glazed surfaces showed no elemental zirconium but instead silicon (Figure 6D) as well as some boron. VUV treatment significantly reduced the atomic percentage of carbon and increased the atomic percentages of oxygen and silicon.

**Figure 7:**
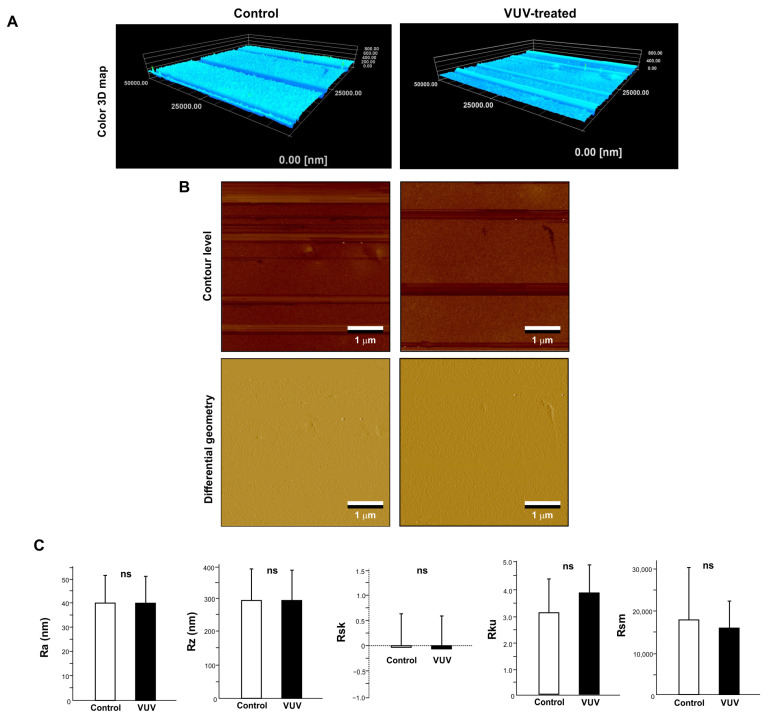
Surface topography of glazed specimens. Three-dimensional color maps (**A**) and contour level and differential geometry two-dimensional (**B**) images of glazed specimens via AFM. (**C**) Histograms of surface roughness parameters calculated via AFM. ns: no significant difference between the control and VUV-treated groups.

Three-dimensional AFM images confirmed that the glazed specimens were smoother than the milled specimens, with no microscale topography and only minor nanoscale roughness (Figure 7A). Contour level and differential two-dimensional geometry images also showed no definable morphology (Figure 7B). There was no difference in the qualitative and quantitative assessments of surface morphology/topography between the control and VUV-treated specimens (Figure 7A–C).

**Figure 8:**
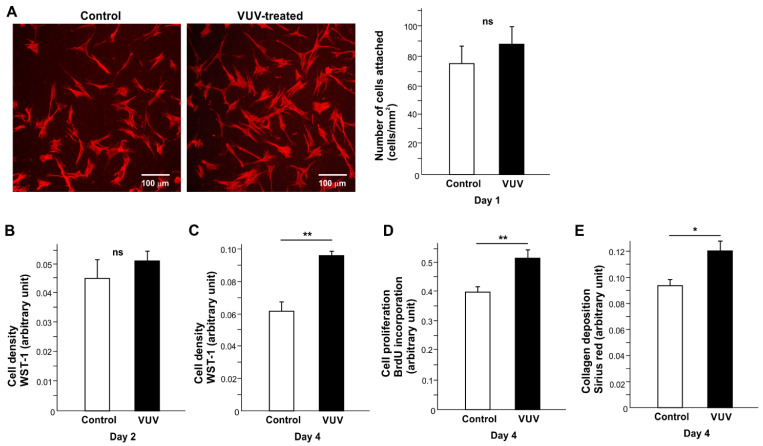
Fibroblast behavior and function on glazed specimens with or without VUV treatment. (**A**) The number of fibroblasts attaching to specimens during the first day of culture. Representative fluorescence microscopic images and the number of attached cells counted on the images are shown. Cell density evaluated via the WST-1 assay on days 2 (**B**) and 4 (**C**). (**D**) Cell proliferation evaluated by BrdU incorporation into DNA on day 4. (**E**) Collagen deposition evaluated on day 4 and the measured metrics. * *p*  <  0.05, ** *p*  <  0.01, statistically different between the control and VUV-treated groups. ns: not significant.

With respect to the biological effects of glazed zirconia on oral fibroblasts, cell density on day 4, BrdU incorporation into DNA, and collagen production were all significantly increased on VUV-treated specimens, although the initial behavior of cells, such as the number of cells attaching on day 1 and cell density on day 2, was not affected (Figure 8A–E).

## 4. Discussion

Here, we show, for the first time, the horizontal transmigration of human oral fibroblasts exclusively on VUV-treated zirconia surfaces, revealing a novel functionalization of biomaterials using VUV light. Gingival soft tissue adhesion and sealing around dental implants, related prosthetic components, and crowns are a clinical imperative to prevent periimplantitis; thus, the observation of robust, VUV-induced migration of fibroblasts is of clinical significance. Also of note, there was transmigration across a specimen gap of 150 µm, which is likely to be of clinical advantage since gaps exist between natural teeth and crowns, dental implants and prosthetic components. VUV-treated zirconia surfaces may provide a favorable local environment to enable soft tissue adhesion and sealing in the transmucosal or mucosal areas of dental prostheses and restorations.

The across-specimen transmigration phenomenon was supported by the observation of an increased number of cells attaching on day 1 and increased cell density and increased BrdU incorporation into DNA on day 4 on VUV-treated zirconia, suggesting increased cellular recruitment and proliferation. Collagen production was also increased, probably due to the greater number of cells growing on VUV-treated zirconia. We believe that VUV-treated zirconia did not significantly affect the function of individual cells except for their initial attachment and proliferation, given that there was no significant difference in gene expression between control and VUV-treated groups. An increase in cell recruitment and proliferation on biomaterials is often accompanied by accelerated and enhanced cellular spreading during the initial stages of culture, especially immediately after cell seeding of adherent cells such as fibroblasts and osteoblasts [97,98,102,103]. Specifically, cytoplasmic, actin-based structures such as filopodia and lamellipodia are known to extend from the leading edge of migrating cells on UV-treated titanium surfaces [81,104], where they sense the surrounding ECM, cells, and material surfaces to determine the speed and direction of cell movement. Here, cytoplasmic extensions and a narrow stretching of the cellular outline were observed at the leading edge of the migratory zone and even at the leading edge of remote areas on VUV-treated zirconia (Figure 5D,E). Cytomorphometry also revealed increased cellular area, diameter, and perimeter, supporting these qualitative observations and the induced transmigration.

The induced transmigration also helps us to understand the longevity of the VUV photofunctionalization effect, facilitated by our experimental design. For instance, it is well known that the number of cells attaching to UV-treated titanium surfaces, which can be evaluated on day 1 or 2 of culture, is higher than the attachment to control surfaces [67]. However, the cells become confluent at around day five and beyond; thus, the number of cells attaching and proliferating after this time point can no longer be evaluated due to the saturated cell density. Due to cells seeding to the entire substrate surface at the same time, colony and cluster formation occurs from multiple directions, making it difficult to determine whether the UV effect lasts for several days or makes no further contribution beyond the first day or two. Furthermore, these biological effects are thought to be due to the removal of the hydrocarbon pellicle via UV treatment and conversion to superhydrophilicity. Once titanium is exposed to cell culture medium in vitro or blood in vivo, it is unlikely that the pellicle-free, superhydrophilic surface still recruits cells after several days. Unlike conventional cell culture experiments, the across-specimen transmigration assay developed and used here was unique in that it allowed the definition of the start area of migration followed by tracking the subsequent migration mono-directionally onto virgin substrate to up to 6 mm of the original specimen size. Cellular migration is only possible when there is the continuous trigger for cytoplasmic extension and cell proliferation. Our novel culture design testing transmigration provided an opportunity to examine the longitudinal effect of VUV-treated surfaces. Contrary to the hypothetical instant effect limited to the very initial stage after cell seeding, robust on-going transmigration was seen on VUV-treated zirconia even at day 7 of culture, suggesting that VUV treatment exerts a sustainable biological impact for at least for seven days. Furthermore, cytomorphology and cytomorphometry revealed that transmigrated, leading-edge fibroblasts at day seven were enlarged, spindle-shaped cells with highly developed cytoplasmic protrusions on VUV-treated zirconia surfaces. Indeed, the cytomorphometric parameters on day 7 were greater than those on day 1 on VUV-treated surfaces. These findings strongly support a new hypothesis that VUV-induced bioactivated surfaces exert a sustained biological effect. Other materials, including but not limited to titanium, and the behavior of other cell types, such as osteoblasts and mesenchymal stem cells, should be evaluated for possible VUV-inducible transmigration.

The UV light used in this study is new and shows an impressive ability to decompose the hydrocarbon pellicle on titanium and in liquids [88,89]. This UV light is specifically categorized as VUV due to its high energy produced via a short wavelength of 172 nm, in contrast to UVA (400–320 nm), UVB (320–280 nm), and UVC (280–200 nm). The 172 nm wavelength is produced by a xenon excimer lamp, and this wavelength has been shown to induce rapid and maximal pellicle removal in only one minute according to optimization studies [88,89]. The advantage in the pellicle-removing capability of VUV light is explained by its exclusive photochemical decomposition through the production of reactive oxygen species, particularly singlet atomic oxygen, as well as the wider spectrum of direct photophysical decomposition of carbon bonding [88]. The present study is the first to demonstrate the biological impact of VUV treatment of biomaterials and the first to demonstrate that it is possible to decarbonize zirconia surfaces and induce superhydrophilicity after one minute of VUV treatment. Considering that the treatment did not otherwise alter the surface topography, the removal of zirconia pellicle appears to induce the observed changes in fibroblast behavior and consequent transmigration across specimens. Other biological effects of one-minute VUV photofunctionalization, such as its effect on osseointegration around titanium, now need studying.

The effects of UV treatment on zirconia have rarely been explored, with some studies examining the osteoblastic response or simply physicochemical surface changes after treatment via the conventional light sources of UVA and UVC [57,62,105,106]. To explore the generalizability of our findings, this study examined VUV photofunctionalization on another biomaterial, VUV-treated glazed zirconia, where the surface is elemental silicon instead of zirconium. There was increased cell density and collagen production on day 4, indicating an increase in cell proliferation but not attachment on days 1 and 2. These lesser effects than those seen with milled zirconia may be due to the inherently smooth and hydrophilic surface property of glazed specimens, given that the effect of UV photofunctionalization is reduced on smoother surfaces, at least on titanium [67,93]. Nevertheless, VUV photofunctionalization is still effective on zirconium- and silicon-based surfaces, albeit with differences depending on the surface characteristics.

We hypothesized that fibroblast attachment and proliferation would be hindered by tilting the substrates, reasoning that gravity helps cell attachment on horizontally placed specimens but detachment when the substrate and cells are angled. As expected, fibroblast attachment and proliferation were correlated with the angle of specimen tilting, with a considerable (80%) reduction in cell density on 60° specimens. More importantly, compared with the relatively small differences seen between the control and VUV-treated groups in the regular culture setting (0°), obliquely placed specimens amplified the differences and a greater benefit was observed with VUV photofunctionalization on more tilted specimens; indeed, on day 4, the compromised cell density on 45° specimens was fully restored via VUV. Fibroblasts encounter various spatial factors that modify the interaction with biomaterials, and crowns, dental implants, implant abutments, and related components are never flat and horizontal. Given this clinical reality, the accentuated effect of VUV photofunctionalization under challenging conditions was even more clinically significant.

Zirconia specimens were treated using VUV light for 1 min in this study based on the recent verification studies of VUV light [88,89]. The time of VUV treatment necessary to activate specimens to the maximum level has been determined for titanium but not for zirconia. Therefore, more remarkable biological effects could be obtained via longer treatment time than 1 min. An optimization study is required to identify the time of VUV treatment to activate zirconia specimens most effectively. As shown in the increased attachment, initial cell behaviors, and promoted cellular proliferation, no adverse event was found in the study. XPS, SEM and surface profiling also failed to detect a chemical, topographical, or structural change potentially inducing negative side effects. A future study must confirm the harmless effects of VUV treatment of zirconia by examining cell viability. The results of the present study solely imply the biological benefits for the behaviors and responses of fibroblasts. The gingival soft tissue consists of connective tissue fibroblasts and oral epithelial cells [107,108,109,110,111,112,113]; the fibroblasts are derived from the mesenchyme, while the epithelial cells are composed of stratified squamous epithelium. The two cell types differ in shape, behavior, and reaction to external stimuli [114,115,116,117,118,119,120,121,122,123,124,125,126]. Various dental restorations and protheses such as crowns, implant abutments, and tissue bars, are surrounded by both connective tissue and junctional epithelium [115,127,128,129,130,131,132,133,134,135,136]. The present, successful results warrant further studies on epithelial cells for potential synergistic benefits to establish soft tissue seal around zirconia. Another limitation of this study is that VUV-mediated zirconia photofunctionalization is proven for soft tissue cells. The clinical use of zirconia dental implants is on the increase due to its aesthetic advantage and allergy-free properties compared with titanium [2,4,17,18,22,137,138,139,140,141,142,143,144,145,146,147,148,149,150,151,152]. The effects of VUV treatment of zirconia on the behavior and response of osteogenic cells and in vivo osseointegration are urgently desired.

The full range of applications that could benefit from VUV photofunctionalization of zirconia now deserve further consideration. Zirconia is extensively used in modern dentistry as crowns and other restorations as well as implant abutments. Unlike dental implants supplied in a sterile package, those devices are made and finished by hand by dentists and dental technicians and then again, at the chairside, further adjusted and polished, inadvertently contaminating the surfaces with various chemicals, especially carbon-containing molecules. One-minute VUV photofunctionalization as a decarbonizing procedure and biological activator would be of significant clinical benefit, and we saw no evidence of potential adverse events such as discoloration and surface damage.

## 5. Conclusions

This study tested the ability of VUV-treated zirconia to induce unique behaviors in human oral fibroblasts, namely specimen-to-specimen transmigration. Specimen-to-specimen transmigration across a significant distance was exclusively seen with VUV-treated zirconia specimens after seven days of culture, and the process was characterized by enlarged cells with robust cytoplasmic projections at the leading edge. This observation was corroborated by a significant reduction in surface carbon on VUV-treated zirconia and a highly increased attachment and proliferation of fibroblasts on VUV-treated zirconia specimens in the regular cell culture setting. These results provide the first evidence of a sustainable effect of VUV photofunctionalization for at least seven days.

## Data Availability

Data availability on request from the authors.
